# Molecular Approaches to Treating Pediatric Leukemias

**DOI:** 10.3389/fped.2019.00368

**Published:** 2019-09-06

**Authors:** Michaela Kuhlen, Jan-Henning Klusmann, Jessica I. Hoell

**Affiliations:** ^1^Swabian Children's Cancer Center, University Children's Hospital Augsburg, Augsburg, Germany; ^2^Department of Pediatric Hematology and Oncology, Martin Luther University Halle-Wittenberg, Halle, Germany

**Keywords:** leukemia, children, targeted therapy, precision medicine, molecular approaches

## Abstract

Over the past decades, striking progress has been made in the treatment of pediatric leukemia, approaching 90% overall survival in children with acute lymphoblastic leukemia (ALL) and 75% in children with acute myeloid leukemia (AML). This has mainly been achieved through multiagent chemotherapy including CNS prophylaxis and risk-adapted therapy within collaborative clinical trials. However, prognosis in children with refractory or relapsed leukemia remains poor and has not significantly improved despite great efforts. Hence, more effective and less toxic therapies are urgently needed. Our understanding of disease biology, molecular drivers, drug resistance and, thus, the possibility to identify children at high-risk for treatment failure has significantly improved in recent years. Moreover, several new drugs targeting key molecular pathways involved in leukemia development, cell growth, and proliferation have been developed and approved. These striking achievements are linked to the great hope to further improve survival in children with refractory and relapsed leukemia. This review gives an overview on current molecularly targeted therapies in children with leukemia, including kinase, and proteasome inhibitors, epigenetic and enzyme targeting, as well as apoptosis regulators among others.

## Introduction

Leukemia is the most common type of cancer in childhood, accounting for 25–30% of cancers in children and adolescents aged 0–18 years (National Cancer Institute SEER Cancer Stat Facts Annual Report to the Nation 2019, German Childhood Cancer Registry Annual Report 2018). Over the past decades, survival rates have steadily increased and now exceed 90% for acute lymphoblastic leukemia (ALL) and 75% for acute myeloid leukemia (AML) in developed countries (1–5). This success has mainly been achieved through remarkable progress in antileukemic treatment, risk-directed therapy, randomized clinical trials performed by collaborative study groups, supportive care, second-line treatment, and advances in the knowledge of leukemic cell biology including genomic variations (2, 3, 5, 6).

The achievement of improved cure rates and survival into adulthood for most children with leukemia necessitates reduction of acute and long-term toxicity to minimize reduced quality of life, long-term morbidity, and premature death without compromising survival. Yet, even nowadays, leukemia remains the leading cause of death from cancer in children and adolescents in many developed countries. Especially, outcome of refractory/relapsed (r/r) leukemia remains poor (5, 7, 8). Indeed, it becomes more and more difficult to achieve further improvement of survival. This is demonstrated by survival rates of ALL, which seemed to reach a plateau, and the constant non-response/relapse rates despite intensified first-line therapy in AML (1, 5). In addition, the treatment paradigm of even further intensification of traditional multiagent chemotherapy including stem cell transplantation that allowed long-term disease-free survival in childhood leukemia reaches the point of inflection at which as many children decease due to r/r leukemia—and thus chemoresistance—as well as treatment-related toxicity. This underscores the urgent need to identify more effective and less toxic first line and salvage regimens for these patients.

To this end, the ever-expanding knowledge on leukemia biology is vital in identifying novel therapeutic targets by disclosing the heterogeneity of childhood leukemia, by unveiling the molecular drivers and by understanding the mechanisms of drug resistance. These developments may ultimately brake with the practice paradigms of “one-size-fits-all” therapy and guide the development of precision/personalized treatment including immunotherapy and targeted (genomic) therapy to offer the “right drug for the right patient at the right time” even in children (9). As such, treatment of chronic myeloid leukemia (CML) with imatinib (targeting BCR-ABL) is a prime example for precision oncology.

In recent years, several novel subtypes of AML and ALL with various prognostic impact have been identified. These are mainly characterized by genetic alterations that perturb multiple key cellular pathways including hematopoietic development, signaling or proliferation, and epigenetic regulation (10). These alterations partly include actionable genes and may thus serve as therapeutic targets. This progress is chaperoned by a brisk pace in genomic and immunological drug development. To date, several new drugs that may target these alterations have been approved by the European Medicines Agency (EMA) or the United States Food and Drug Administration (US FDA) or are still under development. However, it is hard to keep up with these rapid achievements in busy daily routine.

**Figure 1:**
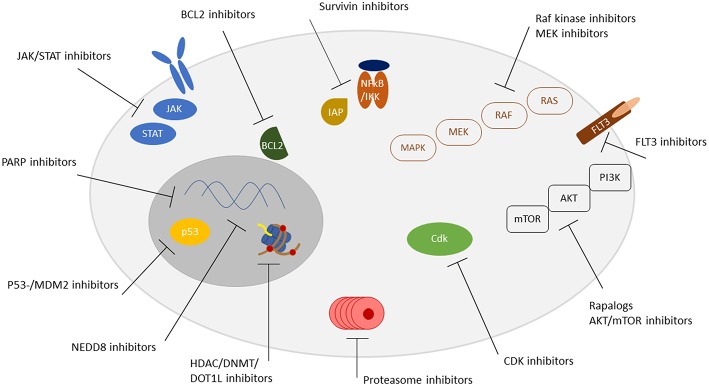
Overview of targetable pathways. Receptors, signaling pathways, and localization of targetable structures are indicated.

Therefore, we herein aimed to give an overview on the most important new drug developments in the treatment of pediatric leukemia. The review focuses on molecular targeted therapies excluding immunotherapy/antibody and CAR-T cell approaches (Figure 1, Table 1).

**Table 1:** Overview of druggable pathways and genetic targets.

**Pathway/mechanism of action**	**(Genetic) target**	**Drug name**	**Adult trials**	**Pediatric trials**	**FLT3-ITD**	**KMT2A-r**	**Ph+**	**IDH1/2**
Kinase inhibition	JAK-STAT pathway	Ruxolitinib	+	+			+	
	FLT3 inhibitor	Midostaurin, quizartinib, lestaurtinib	+	+	+	+		
	MEK inhibitor	Trametinib, selumetinib	+	+				
	Multi-kinase inhibitors	Imatinib, ponatinib, dasatinib, sorafenib	+	+	+		+	
Proteasome/ubiquitin system	Proteasome	Bortezomib, carfilzomib, ixazomib	+	+		+		
	MDM2	Idasanutlin, milademetan, ALRN-6924	+	+	+	(+)	(+)	
	NEDD8	Pevonedistat	+	+				
Epigenetic targeting	HDAC	Vorinostat, panobinostat	+	+				
	DNMT	Azacitidine, decitabine	+	+				
	DOT1L	Pinometostat	+	+		(+)		
Apoptosis	TP53	APR-246	+	No				
	MCL1	S64315	+	No				
	BCL2	navitoclax, venetoclax	+	+		(+)	(+)	+
	survivin	EZN-3042, LY2181308	No	No				
Other approaches	IDH1	Ivosidenib	+	+				+
	CDK4/6	Palbociclib, ribociclib	+	+		+	(+)	
	PARP	Olaparib, veliparib	+	No	+			+
	mTOR	Everolimus, temsirolimus, sirolimus	+	+				
	Menin	MI-463, MI-503, MI-1481, MI-525	No	No		(+)		
	CBFβ-SMMHC	AI-10-49	No	No				

*Drug names are indicated. +, studies currently recruiting (adult trials or pediatric trials, respectively). FLT3-ITD, KMT2A-r, Ph+, IDH1: cases, in which these leukemia specific defects can be targeted by any of the mentioned drugs are designated by “+”*.

## Kinase Inhibitors Across Several Signaling Pathways

More than 500 kinases are encoded in the human genome that are involved in the signal transduction process of the proteome via reversible phosphorylation, thus activating protein function (11, 12). Protein kinases play a major role in cellular regulation including differentiation, survival, proliferation, metabolism, migrating, and signaling, as well as cell-cell interactions. Dysregulated kinases—mainly serine/threonine and tyrosine kinases—are of significant importance in carcinogenesis and metastatic spread (13). Unsurprisingly, previous studies demonstrated that kinases are the most frequently mutated proteins in tumors (14, 15). Moreover, in most malignancies various tyrosine kinases are mutated or overexpressed (16).

The kinome has thereby become an attractive target for the treatment of various human malignancies (11, 12). The first tyrosine kinase inhibitor (TKI), imatinib, was approved for the treatment of CML already in 2001. Generally, kinase inhibitors are employed in those malignancies, which carry specific genetic alterations. To date, 43 orally administered single and multiple kinase inhibitors have been approved and are used in the treatment of a variety of cancers (17). Many more kinase inhibitors are in advanced phase clinical trials and even more in the preclinical stage of drug development.

Most drugs approved to date have limited selectivity thus targeting multiple kinases (18, 19). For example, imatinib shows activity against ABL, BCR-ABL, PDGFR, and c-KIT (20). BCR-ABL kinase selectivity was enhanced with the second-generation TKI nilotinib. The recently approved TKIs bosutinib and ponatinib are designed as dual (SRC-ABL) and multi-kinase (FGFR, PDGFR, SRC, RET, KIT, and FLT1) inhibitors, respectively. Due to potential resistance mechanisms, pharmacokinetics, selectivity and tumor environment, single- and multi-kinase inhibitors have both advantages and disadvantages. In addition, attention should be paid to the various acute and long-term side effects of TKIs including gastrointestinal, cardiovascular, pulmonary, dermatologic, and–particularly in children–endocrine toxicities (21, 22).

Dasatinib has been evaluated in a phase I trial in children and adolescents with imatinib-resistant/-intolerant Philadelphia chromosome (Ph)+ CML, r/r Ph+ ALL and relapsed Ph+ AML (NCT00306202) (23). Based on this, a phase II trial in children with newly diagnosed CML and Ph+ leukemias resistant or intolerant to imatinib (NCT00777036) has been conducted but results have not been published yet. A phase I/II trial for the treatment of r/r leukemias with ponatinib has recently been registered and is not yet recruiting (NCT03934372). The St. Jude trial Total Therapy XVII for Newly Diagnosed Patients With Acute Lymphoblastic Leukemia and Lymphoma (NCT03117751) will give dasatinib to all patients with ABL-class fusions (24).

Other studies evaluate the safety and efficacy of TKI therapy after allogeneic hematopoietic stem cell transplantation (NCT01883219, NCT03624530 both also recruiting adolescents aged 14 years and older) but results are also pending.

### JAK/STAT Inhibitors

The Janus kinase/signal transducers and activators of transcription (JAK/STAT) pathway is the signaling mechanism for cytokines and growth factors and thus plays a key role in cytokine dependent inflammation and immunity. It also affects gene expression via epigenetic modifications (25, 26). JAK/STAT inhibition was expected to suppress the pro-inflammatory tumor-microenvironment and by doing so to provide a strategy for the prevention of tumor progression (27).

Janus kinases (JAK1, 2 and 3) are frequently mutated in a subset of AMLs and high-risk ALLs (28). However, preclinical data of the JAK1/2 inhibitor AZD1480 in patient-derived xenografts (PDXs) from pediatric ALL showed somewhat disappointing results (29). Currently, a phase II trial of the JAK1/2 inhibitor ruxolitinib in combination with standard multi-agent chemotherapy for the treatment of B-precursor ALL in children is running and evaluates the safety and efficacy of this combination (NCT02723994). An already completed phase I/II study of ruxolitinib included patients aged 14 years and older with r/r AML and ALL (NCT01251965). In these heavily pretreated patients, ruxolitinib was reasonably well tolerated with infections being the most frequently reported non-hematologic grade 3 and 4 toxicity (30). Just recently, data from preclinical models provided evidence, that JAK1/2 inhibitors may also be active in pediatric acute megakaryoblastic leukemia (31, 32).

### FLT3 Inhibitors

FMS-like tyrosine kinase 3 (*FLT3*) encodes a class III receptor tyrosine kinase controlling survival, proliferation, and hematopoietic cell differentiation. In up to 20% of pediatric patients with AML, particularly in cytogenetically normal AML, *FLT3* is mutated and confers a poor prognosis (33, 34).

Several non-specific TKIs (e.g., sorafenib, sunitinib) target FLT3 and have been approved for the treatment of a variety of solid malignancies (35). However, they are commonly associated with significant side-effects and toxicity.

The first-in-class FLT3 inhibitor—midostaurin—has been approved for the treatment of adult patients with FLT3-mutated AML. A phase I/II study with single-agent midostaurin (PKC412) in children with r/r acute leukemia including *KMT2A*-rearranged ALL and *FLT3*-mutated AML was terminated early due to insufficient recruitment (NCT00866281). In this heavily pretreated cohort, midostaurin showed limited activity in *FLT3*-mutated AML, whereas in r/r *KMT2A*-rearranged ALL, which is often associated with high-level expression of mutation-negative FLT3 (36), no clinical activity of midostaurin was demonstrated (37). Currently, a phase II trial evaluating midostaurin combined with standard chemotherapy and as single agent post-consolidation therapy is recruiting children with untreated FLT3-mutated AML (NCT03591510).

Another FLT3-inhibitor, quizartinib (AC220), is currently investigated in a phase I/II trial in children with *FLT3*-mutated r/r AML in combination with re-induction chemotherapy and as single-agent maintenance therapy (NCT03793478).

Further phase I/II studies evaluating the safety and efficacy of FLT3-inhibitors (e.g., lestaurtinib) as single-agent therapy or in combination with standard chemotherapy in children with newly diagnosed AML, *KMT2A*-rearranged ALL and r/r AML have already been completed but results have not been published yet (e.g., NCT00469859, NCT00557193).

### MEK Inhibitors

Mitogen-activated protein kinase/ERK kinase (MEK 1/2) inhibitors were the first selective inhibitors of the Ras/Raf/MEK/ERK pathway (also known as MAPK pathway), the latter playing a critical role in the regulation of cell proliferation, differentiation, and survival (38). *MAPK* pathway activating mutations are a hallmark of pediatric low-grade glioma (39) and are highly prevalent in relapsed ALL. Moreover, in relapsed ALL, these mutations are associated with high-risk features and dismal prognosis (40, 41).

To date, only two studies are registered that investigate MEK inhibitors in children with leukemia. In a phase II trial, trametinib is evaluated in children with r/r juvenile myelomonocytic leukemia (JMML) (NCT03190915). Interestingly, following promising preclinical data on a combination of the MEK inhibitor selumetinib and dexamethasone in RAS pathway mutated ALL primagraft cells (42), this combination is now investigated in a phase I/II trial (Seludex trial) in children with r/r RAS pathway mutated ALL (NCT03705507).

To conclude, beside imatinib in the treatment of Ph+ CML and ALL, so far, there is no proof that the use of TKIs—either as single-agent or combination treatment—improves survival in pediatric AML and ALL. Most likely, single small subgroups of children with ALL and AML may benefit from the addition of selected TKIs to conventional chemotherapy and as post-remission/-transplant maintenance, respectively. However, substantial acute toxicity and long-term side effects including hematologic, gastrointestinal, cardiovascular, dermatologic, and endocrine toxicities with varying severity depending on the TKI used need to be considered.

## Ubiquitin-Proteasome System

### Proteasome Inhibitors

The proteasome is a large, multi-subunit protein complex, which is responsible for the degradation of most cellular proteins in physiological conditions, thereby playing a vital role in most cellular processes including cell survival and signaling. As cancer cells have an elevated protein turnover, it was hypothesized early on that they might be sensitive to proteasome inhibition, which indeed turned out to be the case. The first proteasome inhibitor to enter clinical trials was bortezomib, a reversible inhibitor of the 26S subunit (43). Single-agent bortezomib treatment was mostly not efficient. However, the combination with various chemotherapeutic agents proved to be highly beneficial (44). Currently, there are three proteasome inhibitors approved by the FDA, namely bortezomib, carfilzomib, and ixazomib, the latter being the first orally available drug.

The exact molecular consequences of proteasome inhibition via bortezomib are still unsolved, but multiple pathways seem to be involved. One of those results in the stabilization of I-κB, a suppressor of NF-κB signaling, another in the accumulation of the two tumor suppressors p27^KIP1^ and p53 (45, 46).

The TACL study in children with relapsed ALL demonstrated that the combination of bortezomib with vincristine, dexamethasone, doxorubicin, and pegylated asparaginase is highly active in B-precursor ALL but not in T-ALL (47). In using bortezomib, particular attention is needed to infectious complications and peripheral neuropathy. There are currently seven pediatric trials recruiting patients (all ALL) employing bortezomib. All use it in combination with standard chemotherapy backbones. These are AIEOP-BFM ALL 2017 (NCT03643276), International Study for Treatment of High Risk Childhood Relapsed ALL 2010 (NCT03590171), Total Therapy for Infants With Acute Lymphoblastic Leukemia (ALL) I (NCT02553460), Total Therapy XVII for Newly Diagnosed Patients With Acute Lymphoblastic Leukemia and Lymphoma (NCT03117751), ALL-MB 2015 (NCT03390387), a relapse study run by St. Jude Children's Research Hospital (NCT03515200), a relapse study run by the M.D. Anderson Cancer Center (NCT03136146), and a high throughput-guided approach coupled to targeted therapy (NCT02551718, this study also tests carfilzomib). One pediatric AML trial evaluates the combination of bortezomib with sorafenib (NCT01371981).

Carfilzomib, a proteasome inhibitor with fewer off-target effects and supposedly higher degree of inhibition, is tested in one additional pediatric study in r/r ALL (NCT02303821). Two pediatric ALL studies test the orally available ixazomib (NCT03817320, NCT03888534) in a r/r setting.

### MDM2 Inhibitors

*TP53* represents the gene most frequently mutated in human tumors (in some entities up to 80% of cases have heterozygous mutations); mutations are commonly located in the core p53 DNA-binding domain. Wild-type p53 plays central roles in the transcriptional regulation of genes involved in cell-cycle arrest, DNA repair, apoptosis, and senescence. Tumors with wild-type p53 frequently found other ways to block p53 function, such as MDM2 overexpression, which results in p53 inactivation and degradation (48).

Currently, there are two major therapeutic strategies for restoring p53 function in tumor cells, namely increasing the levels of wild-type p53 by preventing its MDM2-mediated degradation. The second approach is to restore p53 transcriptional activity through targeting p53-mutant proteins (see below, section “Targeting Mutant TP53”) (48).

The first small molecule MDM2 inhibitors were nutlin derivatives, which bind the p53-binding cleft of the MDM2 protein. Following exposure to nutlin, cancer cells expressing wild-type p53 (*TP53* mutated cells are intrinsically resistant to this approach) undergo cell cycle arrest and/or apoptosis (48). Interestingly, pre-treatment MDM2 expression can be correlated to clinical response, enabling its use as a biomarker (49).

Available evidence on the efficacy of MDM2 inhibitors in preclinical models of AML as well as clinical studies were recently reviewed (49). There is currently a phase I pediatric trial ongoing, which tests the combination of ALRN-6924 (a dual MDM2/MDMX inhibitor) with cytarabine (NCT03654716) in r/r AML.

Evidence of the therapeutic benefits of MDM2 inhibitors in ALL was also recently reviewed (50). Despite several preclinical and early clinical observations of the efficacy in various poor risk subtypes including Ph+ (51) and *KMT2A*-rearranged ALL (52), there is currently no clinical trial recruiting ALL patients.

### NEDD8 Inhibitor

Neddylation is a posttranslational modification, through which the ubiquitin-like protein NEDD8 is added to lysine residues of substrate proteins. Neddylation is triggered by a cascade of NEDD8-activating enzymes, regulating well known tumor suppressors and oncoproteins, such as VHL, p53, and MDM2 (53).

As such, it does not come as a surprise that neddylation is frequently highly activated in several malignancies and thus presents an attractive therapeutic target. The first NEDD8-activating enzyme (NAE) inhibitor was MLN4924, which functions by blocking the first step of the neddylation cascade. MLN4924 (also known as pevonedistat) has significant tumor-inhibiting effects mainly by triggering apoptosis, senescence and autophagy (54). More recently, there was also preclinical evidence for possibly more potent inhibitors such as TAS4464 (55).

MLN4924 was recently tested in the treatment of AML as NEDD8 regulates the cullin subunits of Cullin-RING ligases (CRLs). These represent the largest family of E3 ubiquitin ligases controlling degradation of about one-fifth of proteasome-regulated proteins and are vital for AML cell survival, among others. Increased neddylation of substrates including cullins promotes degradation of tumor suppressors (e.g., p21 and p27) and facilitates tumorigenesis (54). MLN4924 was indeed shown to have tumor growth inhibiting properties in AML cells *in vitro* and in xenograft assays, later also in clinical studies (56, 57). Currently, there are two studies recruiting pediatric patients, one adding pevonedistat in r/r ALL to a standard backbone ALL chemotherapy regimen (NCT03349281) and the other adding pevonedistat/azacitidine/fludarabine/cytarabine in r/r AML (NCT03813147).

To sum up, bortezomib, one of several inhibitors of the ubiquitin-proteasome system, has already entered phase III clinical trials in children with r/r acute leukemias. However, single-agent treatment was not efficient and whether its combination with conventional chemotherapy improves survival is currently being evaluated. Like any other molecular targeted drug, in combinatorial approaches it comes with considerable side effects including deaths due to infections.

## Epigenetic Targeting

### HDAC Inhibitors

Histone deacetylases (HDACs) are a key component of the epigenetic machinery regulating gene expression. Deacetylation results in a closed chromatin structure and consequently in suppressed transcription of many genes including tumor suppressor genes. HDACs are overexpressed and mutated in tumors, which has led to the hypothesis that they may act as oncogenes (58). In humans, there are 18 HDAC proteins, which are grouped into four classes. HDAC inhibitors block proliferation, induce cell cycle arrest and apoptosis and lead to differentiation. Despite the fact, that HDACs play roles in a vast number of cellular processes (and thus targeting them might come with many off-target effects), they represent desirable therapeutic targets and many HDAC inhibitors have been developed (59). So far, the four pan-HDAC inhibitors vorinostat, romidepsin, bellinostat, and panobinostat have been approved by the FDA. They come with a very similar toxicity profile including gastrointestinal, neurological, and (transient) hematologic toxicities, fatigue and (asymptomatic) ECG changes (60).

Many (pre-)clinical data exist on the use of HDAC inhibitors in leukemias (61). One study showed that pretreatment with vorinostat and/or decitabine induced chemosensitivity in primary pediatric ALL samples (62). Likewise, decitabine and vorinostat followed by re-induction chemotherapy demonstrated a clinical benefit in patients with r/r ALL (patient cohort with a median age of 16 years) (63). In pediatric AML, the combination of azacitidine and panobinostat induced remission in a mouse xenograft model (64). In adult patients, the combination of decitabine plus vorinostat was well tolerated and resulted in a higher response rate (65).

The currently recruiting study NCT02553460 run by the St. Jude Children's Research Hospital tests the addition of bortezomib and vorinostat in infant ALL. Additionally, vorinostat is administered to those childhood T-ALL patients, who showed a poor early response to treatment without a targetable lesion (NCT03117751; Total Therapy XVII study). In pediatric AML, the addition of vorinostat and decitabine to FLAG is also currently evaluated (NCT03263936).

### DNMT Inhibitors

DNA methylation is another major epigenetic modification that impacts nearly all cellular processes. DNA methyltransferases (DNMTs) are overexpressed in several cancer types contributing to tumorigenesis. DNMT3A is mutated in up to 22% of AML patients (66) (its catalytic activity being disrupted by the mutation), but mutations in other genes affecting DNA methylation such as TET1/TET2 have also frequently been described (58).

Azacitidine and decitabine are nucleoside analogous, which are incorporated into DNA resulting in depletion of DNMTs, hypomethylation of DNA, and induction of DNA damage (67). Azacitidine has been successfully used in pediatric MDS (68), common toxicities were hematologic and gastrointestinal.

A phase I clinical trial demonstrated that azacitidine followed by intensive chemotherapy can be used safely to treat children with r/r AML and showed promising activity (69). In T-ALL, first results suggested that decitabine enhances chemosensitivity of both cell lines and patient-derived samples (70).

Treatment with HDAC inhibitors results in increased DNMT1 acetylation and decreased total DNMT1 protein (71). As it has been known for some time that the combination of DNMT and HDAC inhibitors may result in even superior therapeutic outcomes (72), these two drug classes are now frequently combined (for those studies see the section above on HDAC inhibitors). Azacitidine and decitabine are currently compared in a randomized trial in pediatric patients newly diagnosed with AML (NCT03164057). Another trial evaluates the novel approach of treating a molecular AML relapse after first complete remission in pediatric patients (NCT02450877).

### DOT1L Methyltransferase Inhibitor

Disruptor of telomeric-silencing 1-like (DOT1L) is an enzyme, which methylates H3K79. The abnormal expression of KMT2A fusion target genes is associated with high levels of H3K79 methylation at these gene loci (58, 73). Loss-of-function mouse models, as well as small molecular inhibitors of DOT1L, reported that *KMT2A*-rearranged leukemias are DOT1L dependent for proliferation (74).

A recently completed study in adults with advanced *MLL* rearranged acute leukemias showed that the addition of pinometostat, a small molecule inhibitor of DOT1L, was safe with tolerable toxicities including fatigue, nausea, constipation, and febrile neutropenia. Although it has to be noted that this was a phase I study, modest clinical activity could be shown (75). Pinometostat has also been evaluated in a phase I study in children with KMT2A-rearranged r/r leukemias (NCT02141828) but results have not been published yet.

## Apoptosis Regulators

### Targeting Mutant TP53

The general principles of targeting p53 were already detailed above (see section “MDM2 Inhibitors”). As explained, the second approach (besides inhibiting MDM2) involves restoring p53 transcriptional activity (48). Despite being originally considered as undruggable, a small molecule screen nearly 20 years ago identified APR-246 via its ability to induce apoptosis in human tumor cells through restoration of the transactivation function of mutant p53 (76). Since that time, several reports have shown anti-leukemic activity of APR-246 *in vitro* both in ALL (77) and AML (78, 79). A first-in-human trial in refractory hematologic malignancies concluded that APR-246 could be administered safely and induced p53-dependent biologic effects in tumor cells *in vivo* (80).

Currently, there are only studies recruiting, which test APR-246 plus azacitidine in adults with TP53 mutant MDS/MPN/CMML/AML plus or minus allogeneic stem cell transplantation. Unpublished results of the latter (abstract Blood 2018 132:3091) show that this combination is well tolerated. Responses have been achieved in all evaluable pts (82% CR) accompanied by deep molecular and durable remissions. In April 2019, a fast track and orphan drug designation was granted by the FDA for APR-246 in *TP53*-mutated MDS.

In summary, more studies with molecules targeting mutant p53 (APR-246 being the only one, which has been employed in hematologic malignancies) are needed, as it is not yet clear, which of the nearly 1,500 different missense mutations they are able to target (48) but first results are encouraging.

### BCL2 Inhibitors

The oncogenic protein B-cell lymphoma 2 (BCL2) was discovered in B-cell leukemias and follicular lymphomas 40 years ago. BCL2 induces transformation by blocking apoptosis. Later, many structurally related proteins were identified, which are clustered into three groups: (i) multidomain anti-apoptotic proteins such as BCL2, BCL-XL and MCL1, (ii) multidomain pro-apoptotic effector proteins such as BAX or BAK and (iii) BH3-only group of pro-apoptotic proteins (including BIM, BID, PUMA, BAD, BIK, NOXA) (49).

Members of the BCL-2 gene family play vital roles in apoptosis by controlling pro-apoptotic and anti-apoptotic intracellular signals (81). Inhibitors targeting both MCL1 and BCL2 have been developed.

Navitoclax, a first generation BCL-2 inhibitor targeting BCL-2 and BCL-XL, showed clinical activity, however, its use was restricted by the occurrence of neutropenia. Shortly after, the selective BCL-2 inhibitor venetoclax was developed and approved by FDA/EMA for a subset of CLL patients. In acute leukemias, venetoclax has shown efficacy both in AML and in ALL. In using venetoclax, attention is needed to tumor lysis syndrome, other side effects include hematologic and gastrointestinal toxicity, respiratory infections, and fatigue (82).

Of special interest in the pediatric setting was a report, which showed that patient-derived ALL cells carrying the *KMT2A-AF4* fusion had high BCL-2 levels (via H3K79 methylation through DOT1L) and were highly sensitive to treatment with venetoclax, also in a xenograft setting (83).

Moreover, the combination of dasatinib and venetoclax resulted in superior antileukemic efficacy compared to either agent alone in Ph+ ALL xenografts (84). Another report showed that the combination of venetoclax and the JNK inhibitor SP600125 exhibited synergistic cytotoxicity against imatinib-resistant Ph+ ALL cells (85).

Several recruiting studies test the combination of venetoclax with cytarabine and daunorubicine liposome (NCT03826992), with cytarabine with or without idarubicin (NCT03194932), and with navitoclax and chemotherapy (NCT0318126) in children with r r/r ALL, AML and acute leukemia of ambiguous lineage, respectively, to name just a few. In addition, one study run by the MD Anderson Cancer Center including children is testing venetoclax in combination with chemotherapy including nelarabine in previously untreated patients with T-ALL and lymphoblastic lymphoma (NCT00501826). Noteworthy, a phase I/II study has just recently been registered but is not yet recruiting which will evaluate the MDM2 antagonist idasanutlin in combination with either chemotherapy or venetoclax in children and young adults with r/r acute leukemias (NCT04029688).

### Survivin Inhibitors

*Survivin* (*BIRC5*) is a member of the inhibitor-of-apoptosis proteins (IAPs) family. It is an oncofetal protein, which is not expressed in differentiated normal tissue. Furthermore, survivin overexpression has been correlated with resistant and refractory disease in many different malignancies. IAPs regulate caspase activity, cell division, and cell survival pathways as well as being involved in DNA repair and drug resistance (86).

An early report showed that an imatinib-resistant Ph+ chronic myelogenous leukemia (CML) cell line had high survivin expression levels. Down-regulating survivin expression induced cell-growth arrest and subsequent cell death (87). Later, high survivin expression levels were also reported in ALL and downregulation of survivin via the antisense oligonucleotide EZN-3042 in combination with chemotherapy resulted in deep molecular remission of disease in a xenograft model (88). Later, this drug was employed in a trial with pediatric r/r ALL. However, the combination of EZN-3042 with intensive reinduction chemotherapy led to intolerable toxicity (grade 3 gamma-glutamyl transferase elevation and gastrointestinal bleeding) and the trial was terminated (89).

Another survivin antisense oligonucleotide, LY2181308, was tested in an adult phase I refractory/relapsed AML study, both as a monotherapy and in combination with cytarabine and idarubicine. In this case, the drug was well tolerated and showed some clinical benefit, which will need to be verified in future clinical trials (90).

In summary, despite the growing knowledge on survivin and despite its important roles in oncogenesis, the development of survivin inhibitors or survivin-related molecular therapies has been slow (86). Currently, there are no trials listed employing survivin inhibitors.

## Other Targeted Approaches

### Targeting Metabolic Enzymes

Two isoforms of isocitrate dehydrogenase (IDH), *IDH1* and *IDH2*, are among the most commonly mutated genes in AML occurring in about 20% of all newly diagnosed patients. They are key enzymes in the metabolism of a cell and also function in the regulation of oxidative stress. The heterozygous, mutually exclusive mutations occur at hotspot positions (*IDH1*^*R*132^, *IDH2*^*R*140^, *IDH2*^*R*172^) and lead to a neomorphic enzyme activity resulting in the generation of very high levels of 2-hydroxyglutarate. This oncometabolite causes epigenetic changes and impairs cell differentiation. Preclinical data indicated early on that targeted inhibition of both IDH1 and IDH2 blocked colony formation of AML cells from *IDH1*-mutated patients and induced differentiation (91, 92).

As there is great excitement regarding this class of inhibitors, many studies testing these substances both as monotherapies as well as in addition to standard chemotherapy backbones are being conducted, however, not yet in a pediatric setting, where *IDH1/2* mutations are rare. Only ivosidenib, a small-molecule IDH1 inhibitor approved by the FDA in 2018, is offered in an expanded access program in r/r AML with an *IDH1*^*R*132^ mutation to children ≥12 years of age (NCT03245424).

### Cell Cycle Regulation

Loss of cell cycle control resulting in unrestrained growth is generally considered a hallmark of cancer and aberrations in the cyclin-dependent kinase-retinoblastoma (CDK-Rb) pathway are common in multiple malignancies. Consequently, inhibition of this pathway is an attractive therapeutic strategy. The G1 cyclin-dependent kinases 4 and 6 (CDK4 and 6) phosphorylate—in a complex with cyclin D—retinoblastoma protein (Rb), which leads to cell cycle progression and cell growth (93).

More than 10 years ago, it was reported that the D-cyclin-CDK4/6 complex was a downstream effector of FLT3-ITD signaling. Inhibiting CDK4/6 caused sustained cell-cycle arrest (94). Another study showed that the CDK4/CDK6 kinase inhibitor palbociclib (PD 0332991) sensitized AML cells to cytarabine, opening the possibility of a combination therapy (95).

Ribociclib, another CDK4/CDK6 kinase inhibitor, enhanced glucocorticoid sensitivity in primary cultures derived from bone marrow of pediatric B-precursor ALL patients (96). A recent study showed that palbociclib suppressed dissemination of Ph+ ALL and prolonged survival in a xenograft model (97).

Of special interest in the pediatric setting was the observation, that the KMT2A fusion proteins activate CDK6, thus driving proliferation in *KMT2A*-rearranged infant ALL. Treating *KMT2A*-rearranged leukemia cell lines with palbociclib resulted in a G1 arrest in ALL (98).

The high interest in this class of drugs is reflected by a total of three studies recruiting pediatric patients, namely one, which evaluates ribociclib/everolimus/dexamethasone in relapsed ALL (NCT03740334), and two combining palbociclib with mostly standard chemotherapy backbones in r/r ALL (NCT03515200, NCT03792256).

The various CDK4/6 inhibitors show similar side effects including high-grade hematologic toxicities, gastrointestinal and hepatobiliary toxicities, and QTc prolongation.

### PARP Inhibitors

Several mechanisms seem to be responsible for the action of poly (ADP-ribose) polymerase (PARP) inhibitors, one is synthetic lethality. The PARP inhibitor blocks base excision repair leading to a double strand break. As tumor cells frequently have defects in homologous repair genes—such as *BRCA1, BRCA2, ATM*, or Fanconi anemia pathway mutations (which on their own are advantageous and result in growth advantages through increased genomic instability)—it will be unable to repair the double strand defect and will undergo apoptosis (99). This makes the concept of PARP inhibitors attractive, as they target cancer cells based on their genetic deficiencies while sparing normal cells, which have backup mechanisms for repairing DNA strand breaks.

Preclinical evidence of the potency of PARP inhibitors in leukemia included anti-proliferative effects in T-ALL cell lines (100) and re-sensitizing adriamycin-resistant leukemia cells (101). Recently, it was hypothesized, that *TET2*, which is frequently mutated in hematologic malignancies, maintains genomic stability via promotion of DNA damage repair and that loss of *TET2* might sensitize myeloid leukemia cells to PARP inhibitors (102).

As mentioned above, FLT3-ITDs, which can be found in up to 23% of AML patients, confer a poor prognosis. It was shown that treatment with a FLT3 inhibitor caused downregulation of DNA repair proteins. The combination with a PARP inhibitor significantly delayed disease onset and reduced leukemia-initiating cells in a FLT3-ITD-positive primary AML xenograft mouse model (103).

Many PARP inhibitors have been developed, of which two (olaparib and veliparib) have been tested in acute leukemias. A previous trial reported that veliparib/temozolomide was well tolerated and showed activity in advanced AML (104). Several current trials are exploring PARP inhibitors, however only in the adult setting.

### mTOR Inhibitors

The PI3K/Akt/mTOR pathway is a key regulatory pathway, which controls cell growth, survival, and cellular metabolism. The discovery of high mutational frequencies in multiple malignancies of both genes in the pathway itself but also in upstream, membrane-associated genes, early on sparked interest in targeting this pathway therapeutically (105). Upon mutation, this pathway becomes constitutively active in several malignancies, including ALL and AML (106). In some solid malignancies such as breast cancer and renal cell carcinoma, mTOR inhibitors are already firmly established in therapeutic regimens (107).

Early studies in leukemia showed that allosteric mTOR inhibitors resulted in decreased growth and induced apoptosis in ALL cell lines as well as xenograft models (108). Mammalian target of rapamycin (mTOR) exists in two complexes, mTORC1 and mTORC2. The rapamycin analogs (rapalogs) everolimus, temsirolimus and sirolimus, which target mTORC1 through binding to the protein FKBP12 (109), were the first mTOR inhibitors to enter clinical trials.

Several studies have been performed so far with either rapalogs as monotherapy or as an addition to established therapies. However, the addition of temsirolimus to UKALL R3 in children with second or greater relapse of ALL resulted in excessive toxicity including mucositis, ulceration, hypertension with reversible leukoencephalopathy, liver toxicity and sepsis. Moreover, despite the fact, that an inhibition of PI3K signaling was detected in all patients at an early timepoint, there was no correlation with clinical responses at the end of re-induction therapy (110). Many more studies have been conducted in leukemias [for an overview see Fransecky et al. (105)] but mostly with only modest clinical responses.

A recent phase I trial tested co-administering everolimus with a four-drug reinduction in children with ALL and came to the conclusion that this treatment was well tolerated and was associated with favorable CR2 rates (111). However, only 22 patients were enrolled.

Currently, there are several studies recruiting, which are exploring temsirolimus (NCT01614197), everolimus (NCT03740334) and other rapalogs and some, for which recruitment was terminated but for which results are still pending.

In summary, given the initially convincing preclinical data on mTOR inhibition in acute leukemias, the results of nearly all clinical trials have not lived up to the expectations. Yet, there is still much interest in targeting this pathway and novel inhibitors are still being developed for use in future studies (105).

### Menin Inhibitors

Menin, encoded by the *MEN1* gene, is considered an oncogenic cofactor of KMT2A fusion proteins, as it functions as an adaptor between KMT2A and LEDGF. It was shown that its presence is required for transformation of *KMT2A*-rearranged leukemia (112, 113).

As it represents an attractive therapeutic target, a whole series of small molecule inhibitors [i.e., MI-463 and MI-503 (114)] were developed, which block the KMT2A-binding site on Menin in a fusion-partner independent manner. This led to a downregulation of KMT2A-fusion targets, differentiation of leukemic blasts, and prolonged survival of mouse models of *KMT2A*-rearranged leukemia (114, 115). Recently, two more menin inhibitors were reported [MI-1481 (116) and M-525 (117)]. One group reported that the combination of a menin inhibitor with a DOT1L inhibitor proved beneficial as it facilitated enhanced induction of differentiation and apoptosis in a mouse model injected with *KMT2A-AF9* leukemic cells (118).

However, evidence on menin inhibitors has so far only been preclinical, as none of those compounds has entered clinical trials yet.

### Targeting the CBFβ-SMMHC Rearrangement

AML is characterized by recurrent chromosomal rearrangements encoding for fusion proteins that both initiate and maintain leukemic cell growth. One such rearrangement is inv (16) (p13q22) resulting in the CBFβ-SMMHC fusion protein. This transcription factor fusion outcompetes wild-type CBFβ for binding to the transcription factor RUNX1, neutralizes RUNX1-mediated repression of MYC expression, and induces AML. A small molecule protein-protein interaction inhibitor, AI-10-49, was developed, which selectively binds to CBFβ-SMMHC and disrupts its binding to RUNX1 (119, 120). Treatment of primary inv (16) AML patient blasts with AI-10-49 restored RUNX1 transcriptional activity and delayed leukemia progression in mice (121). However, AI-10-49 has not entered clinical trials so far.

## Conclusion and Outlook

The concept of contemporary randomized clinical trial (RCT) designs providing risk-adapted therapy has significantly contributed to the success story of acute leukemia in childhood. To date, the gold standard in leukemia treatment is multiagent chemotherapy, in some cases including hematopoietic stem cell transplantation, within traditional RCTs. Recent advances in cancer genomics, knowledge on stem cell biology, and experimental modeling of leukemia provide new insights into leukemogenesis, subtypes of leukemia, drug resistance, host pharmacogenetics, and potential therapeutic approaches as well as targeted therapies. In addition, a wealth of new cancer drugs has been approved by the EMA and US FDA and most likely much more will be approved in the near future.

A number of the recently approved new cancer drugs have already been evaluated in children with leukemia. However, except for rare examples such as imatinib, groundbreaking improvements in survival have not been achieved. Instead, most of them failed to demonstrate sufficient single-agent activity. For instance, employing 53 approved new cancer drugs (across several cancer types not only hematologic malignancies), only a mean total increase in overall survival of 3.4 months over the so far available treatments was seen in adults (122). Thus, the great hopes accompanied by precision medicine have -at least in adults- not been fulfilled so far. Yet, the impact of targeted therapies on improving survival in children with cancer still remains to elucidated by the range of pediatric precision oncology trials which are now available.

Most likely, optimal (personalized) treatment strategies in children and adolescents with acute leukemias will have to integrate traditional chemotherapy, immunotherapy, and molecularly targeted drugs as well as combinations of those to obtain synergistic effects. Noteworthy, currently, all different types of immunotherapies are prioritized, in, at least, ALL.

## Author Contributions

MK and JH wrote the manuscript. J-HK revised the manuscript critically for important intellectual content. All authors contributed to manuscript revision, read and approved the submitted version.

### Conflict of Interest Statement

The authors declare that the research was conducted in the absence of any commercial or financial relationships that could be construed as a potential conflict of interest.
